# Regulatory T Cells: Barriers of Immune Infiltration Into the Tumor Microenvironment

**DOI:** 10.3389/fimmu.2021.702726

**Published:** 2021-06-10

**Authors:** Ellen N. Scott, Angela M. Gocher, Creg J. Workman, Dario A. A. Vignali

**Affiliations:** ^1^ Department of Immunology, University of Pittsburgh School of Medicine, Pittsburgh, PA, United States; ^2^ Tumor Microenvironment Center, University of Pittsburgh Medical Center (UPMC) Hillman Cancer Center, Pittsburgh, PA, United States; ^3^ Graduate Program of Microbiology and Immunology, University of Pittsburgh School of Medicine, Pittsburgh, PA, United States; ^4^ Cancer Immunology and Immunotherapy Program, UPMC Hillman Cancer Center, Pittsburgh, PA, United States

**Keywords:** regulatory T cells (Treg), immune infiltration, tumor microenvironment, cancer, vasculature, stroma

## Abstract

Regulatory T cells (T_regs_) are key immunosuppressive cells that promote tumor growth by hindering the effector immune response. T_regs_ utilize multiple suppressive mechanisms to inhibit pro-inflammatory responses within the tumor microenvironment (TME) by inhibition of effector function and immune cell migration, secretion of inhibitory cytokines, metabolic disruption and promotion of metastasis. In turn, T_regs_ are being targeted in the clinic either alone or in combination with other immunotherapies, in efforts to overcome the immunosuppressive TME and increase anti-tumor effects. However, it is now appreciated that T_regs_ not only suppress cells intratumorally *via* direct engagement, but also serve as key interactors in the peritumor, stroma, vasculature and lymphatics to limit anti-tumor immune responses prior to tumor infiltration. We will review the suppressive mechanisms that T_regs_ utilize to alter immune and non-immune cells outside and within the TME and discuss how these mechanisms collectively allow T_regs_ to create and promote a physical and biological barrier, resulting in an immune-excluded or limited tumor microenvironment.

## Introduction

Regulatory T cells (T_regs_) are suppressive CD4^+^ T cells that are characterized, and largely regulated, by expression of the master transcription factor, forkhead box protein 3 (FoxP3) (1). T_regs_ are critical in the maintenance of peripheral tolerance to prevent autoimmune disease. During pathogenic insults, T_regs_ prevent overt immune activation in efforts to limit tissue damage. T_regs_ are also found in tumors with the ratio of T_regs_ to T cells positively correlating with poor prognosis and response to immunotherapy (2, 3). Strikingly, T_reg_ depletion in murine tumor models results in complete tumor clearance, however these mice ultimately succumb to lethal systemic autoimmune disease (4–7). The drastic effect of T_regs_ on tumor growth has sparked interest in elucidating T_reg_ function within the tumor microenvironment (TME) in efforts to selectively target tumor-infiltrating T_regs_ while sparing peripheral T_regs_ (8).

Immunotherapies designed to target intratumoral Tregs have focused on key surface markers that are highly expressed and contribute to their suppressive functions, such as CTLA-4, CD25, TIGIT, 4-1BB, OX-40, CCR4, and CCR8. Targeting these markers therapeutically has had some clinical success. The first FDA-approved immunotherapy utilized a blocking monoclonal antibody specific for cytotoxic T-lymphocyte-associated protein 4 (CTLA-4 or CD152) (ipilimumab), which preserves T cell activation *via* preventing CTLA-4 binding to CD28 thus allowing for CD28 engagement of CD80/86 (9). Currently, the complete mechanism for ipilimumab is not fully elucidated but may also involve depletion of T_regs_ *via* antibody-dependent cell-mediated cytotoxicity (ADCC) (10). Despite ipilimumab prolonging patient survival and increasing the five-year survival rate, 10-15% of patients experience Grade 3-4 immune-related adverse events, thus investigation of additional T_reg_-targeting strategies are warranted (11). Monoclonal antibodies against CD25, OX-40 and GITR have produced favorable anti-tumor effects, which were dependent on ADCC mediated T_reg_-depletion (12). Studies to uncover both novel molecules enriched on tumor infiltrating T_regs_ or mechanisms of suppression unique to the TME are warranted to improve targeted immunotherapy while limiting toxicity.

**Figure 1:**
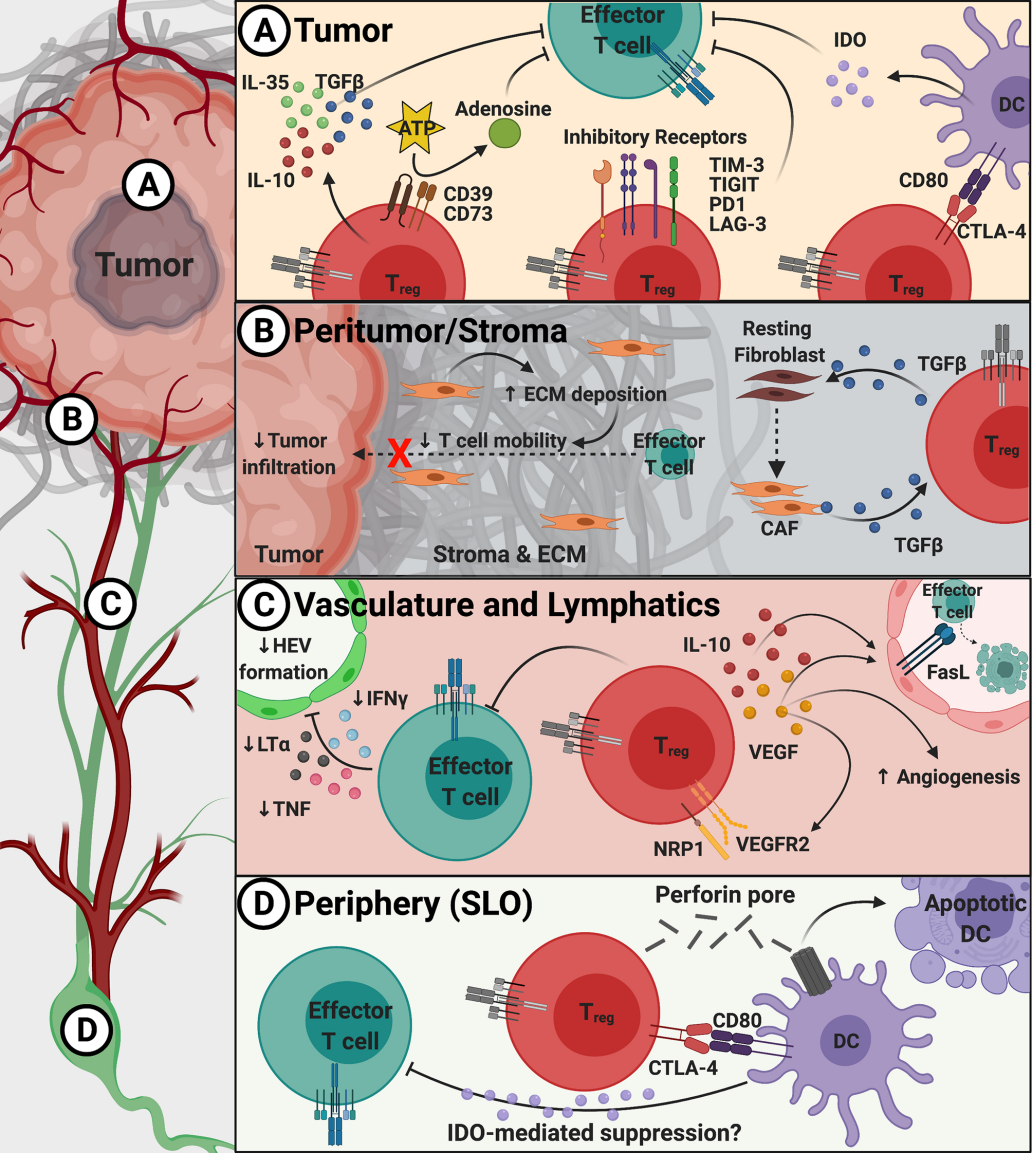
Overview of suppressive mechanisms used by T_regs_ to create barriers to immune infiltration into tumors. Panel **(A)** Within the TME, T_regs_ utilize inhibitory receptors (TIM-3, TIGIT, PD1, and LAG-3), inhibitory cytokines (TGFβ, IL-10, and IL-35), DC modulation (via CTLA-4 and LAG-3), and metabolic disruption (via CD39/CD73) to suppress the anti-tumor T cell response. **(B)** T_reg_-derived TGFβ induces cancer-associated fibroblast (CAF) development that increases extracellular matrix (ECM) production and deposition within the peritumoral space (stroma) to inhibit effector T cell migration. **(C)** T_regs_ block entry of effector T cells through preventing proper cytokine signals that promote high endothelial venule (HEV) formation as well as production of inhibitory IL-10 and VEGF to promote dysregulated angiogenesis. **(D)** In the periphery and secondary lymphoid organs (SLO), T_regs_ can modulate DC maturity and induce apoptosis to prevent proper effector T cell activation.

T_regs_ are found throughout the TME and can even exert suppressive function at a distance, forming physical, metabolic, and trafficking ‘barriers’ to exclude pro-inflammatory cells from the TME. These barriers can be both ‘physical’, by limiting the ability of effector T cells to enter into the tumor, and ‘functional’, by limiting the activity of effector cells already within the TME. Together, these barriers create an immune-excluded TME with studies showing that decreased CD8^+^ T cells, specifically, within the vicinity of tumor cells correlates with poor outcomes (13). The primary ‘barriers’ constructed by T_regs_ that prevent the infiltration of pro-inflammatory cells include poor activation of T cells in the periphery, disorganized vasculature, prevention of the formation of lymphatic structures in the TME and a stroma that hinders the migration of cells into and around the tumor bed (14, 15). These barriers of immune exclusion that T_regs_ erect will be discussed herein, starting with the tumor core and working outward through the peri-tumor to the stroma, ending with lymphatic structures and the periphery (**Figure 1**). Investigation of the pro-tumorigenic effects of T_regs_ in the whole tumor (non-micro) environment is necessary to elucidate novel therapeutic strategies to dismantle pro-tumor T_regs_ while maintaining peripheral tolerance.

## T_regs_ as Anti-Inflammatory Intratumoral Barriers

The frequency and organization of T_regs_ within the TME is diverse in cancer patients; however, high T_reg_ infiltration often correlates with poor prognosis in many cancer types (16–18). The origin of these T_regs_ – either thymically (tT_regs_) or peripherally-derived (pT_regs_) – is still being debated (19). T cell receptor (TCR) sequencing studies in carcinogen-induced murine models and in human melanoma, gastrointestinal and ovarian cancers have shown distinct TCR sequences between intratumoral T_regs_ and FOXP3^–^ conventional CD4^+^ T cells (20–22). A study using a genetically-driven prostate cancer murine model showed that intratumoral T_regs_ were thymically-derived, had less diverse TCRs, and had TCRs specific for the prostate tissue (23). Conversely, a recent study in breast cancer patients showed 65% TCR overlap of intratumoral T_regs_ with activated conventional CD4^+^ T cells (24). Overall, T_reg_ conversion in the periphery and upon entry into the TME may be a rare event and may only be observed with the use of TCR transgenic mice or human tumors of specific tissue origins. However, having a TME that contains pT_regs_ and/or tT_regs_ may provide diverse functions (stability, effector and cytokine profile) that may provide a therapeutic opportunity to dedifferentiate T_regs_ to an unstable, non-immunosuppressive state (ex-T_regs_) (25).

Tumors create an immunosuppressive environment that attracts T_regs_ and also support their anti-tumor function. Tumors secrete the CC chemokine ligand 22 (CCL22) and CCL17, which recruit T_regs_ to the tumor *via* T_reg_ expression of the CC chemokine receptor 4 (CCR4) (26). Use of mogamulizumab (anti-CCR4) in patients with cutaneous T cell lymphoma or solid tumors, reduced the levels of circulating or intratumoral CCR4^+^ T_regs,_ respectively, but did not induce potent antitumor effects (27, 28). Combination of mogamulizumab with nivolumab (anti-PD1) in phase I clinical studies was tolerable and increased intratumoral CD8^+^ T cells and decreased T_regs_ in patients with solid tumors, making this therapeutic combination an effective option (29). Under hypoxic conditions, tumors secret CCL28 which recruits T_regs_ *via* CCR10 (30). Additionally, tumors secrete CCL5 which recruits T_regs_ *via* CCR5 and pre-clinical studies with CCR5 inhibitors have decreased T_reg_ tumor infiltration and tumor growth (31, 32).

Conventionally, T_regs_ have higher affinity to self-antigen compared to other T cells which allows for suppression of autoreactive T cells and prevention of autoimmune disease. Tumors express self-antigens that are over expressed, inappropriately expressed, or mutated and preferentially promotes the activation and sequestration of T_regs_ as seen by the expansion of a few T_reg_ clones specific for tumor antigens in cancer patients (33–35). A study using non-TCR transgenic mice showed that the TCRs of intratumoral T_regs_ are also found on T_regs_ from tumor draining lymph nodes (dLN), suggesting that T_regs_ are activated in the dLN, clonally expand, and migrate to the tumor where they accumulate (36). Although these data strongly suggest that T_regs_ recognize specific tumor antigens, albeit lower diversity compared to activated intratumoral conventional CD4^+^ cells, not all T_regs_ in the TME have tumor antigen-specific TCRs.

The high proliferation index of cancer cells creates a high energy demand, which forces the tumor to switch from oxidative phosphorylation to glycolysis (referred to as the Warburg effect), which generates a lactic acid-rich, glucose-poor, and hypoxic TME (37). Uptake of glucose by intratumoral T_regs_ promotes instability and loss of suppressive function. Instead, intratumoral T_regs_ upregulate pathways involved in lactic acid metabolism, and lactate uptake is required for maintenance of suppressive function of intratumoral, but not peripheral, T_regs_ (38). Mechanistically, Foxp3 promotes glycolysis *via* binding to the promoter of Myc and inducing expression (39). Deletion of hypoxia-inducible factor 2α (HIF-2α) from murine T_regs_ destabilized T_regs_ and prevented growth of MC38 colon adenocarcinoma (40). Collectively, consumption of glucose and oxygen by the proliferating tumor constructs a favorable metabolic landscape for T_regs_ to stably thrive in the TME.

Once in the tumor, T_regs_ suppress the anti-tumor response through contact-dependent and contact-independent mechanisms. Contact-dependent mechanisms utilizing CTLA-4, lymphocyte-activation gene 3 (LAG-3), and T cell immunoglobulin and ITIM domain (TIGIT) prevent activation and maturation of dendritic cells (DCs) thus preventing an effective anti-tumor T cell response (**Figure 1A**). CTLA-4 on T_regs_ binds CD80 molecules on DCs to induce transendocytosis and downregulation of CD80 expression and production of the inhibitory molecule indoleamine 2,3-dioxygenase (IDO) (41–44). While the intracellular domain of CTLA-4 is not thought to have a signaling function, it is important for the regulation of endocytosis and trafficking (45, 46). Specifically, a mouse model of T_reg_-specific CTLA-4 deletion resulted in fatal lymphoproliferative and autoimmune diseases while drastically limiting tumor progression, illustrating the importance of CTLA-4 in mediating T_reg_ function through transendocytosis of CD80 and CD86 (44, 47, 48). LAG-3 binding to major histocompatibility complex class II on DCs reduces the expression levels of the costimulatory molecule CD86 and IL-12 cytokine production (49). TIGIT ligation of CD155 on DCs increased production of IL-10 and lowered IL-2, supporting an immunosuppressive environment (50, 51). While programmed cell death 1 (PD1) and T-cell immunoglobulin and mucin-domain containing-3 (TIM-3) are highly expressed on T_regs_ and important for suppressive function, the mechanisms are unknown (52, 53).

Contact-independent mechanisms of T_regs_ include the secretion of the inhibitory cytokines IL-10, IL-35, and transforming growth factor-β (TGFβ), which suppress the activity of effector cells (**Figure 1A**). IL-10 suppresses *via* inhibition of CD28 tyrosine phosphorylation and induction of CD8^+^ T cell exhaustion *via* upregulation of B lymphocyte-induced maturation protein-1 (BLIMP1) (54, 55). IL-35 limits the proliferation and memory formation, and promotes exhaustion in CD8^+^ T cells similarly to IL-10 by expression of BLIMP1 and downstream inhibitory receptors (54, 56). TGFβ decreases effector function *via* inhibiting the transcription of proinflammatory cytokines (interferon gamma [IFNγ]) and granzyme B, and T helper cell transcription factors (T-box transcription factor and GATA binding protein 3), although the precise mechanism of action remains unknown (57–60). While these activities represent the general role of TGFβ, it is important to understand that different isoforms may have differing functions based on the expression pattern in various cancers (61–63). Thus, secretion of these cytokines by T_regs_ acts as a functional ‘barrier’ that prevents the function and expansion of surrounding effector T cells.

T_regs_ in the TME also suppress anti-tumor immunity through metabolic disruption *via* CD25/IL-2, CD39/CD73, and IDO (**Figure 1A**). IL-2 is required for effector T cell differentiation and fate upon immune activation and is critical for the development, regulation, proliferation and maintenance of T_regs_ (64). T_regs_ express high levels of the IL-2 receptor, CD25, which also deprives surrounding effector T cells of IL-2 (65). T_reg_ expression of the ectonucleotidases CD39 and CD73 convert ATP and ADP into adenosine, which suppresses effector T cells *via* the adenosine receptor 2A (66, 67). Interestingly, T_reg_ ligation of CD80/CD86 on dendritic cells (DCs) *via* CTLA-4, increases the production of IDO (47) (**Figure 1A**). IDO metabolizes the essential amino acid tryptophan, limiting its availability, into different suppressive metabolites including kynurenine which inhibits T cell proliferation (43, 68). Despite promising findings in murine models and human *in vitro* studies, a Phase III clinical study with the IDO1 inhibitor epacadostat in combination with pembrolizumab (anti-PD1) in melanoma was disappointing (69). The lack of epacadostat efficacy in the clinic may be due to low initial levels of tryptophan and kynurenine in the TME, the presence of other enzymes able to catabolize tryptophan such as IDO2 and tryptophan 2,3-dioxygenase (TDO2), inefficient inhibition of IDO1, or adaptive resistance.

Through the expression of inhibitory receptors, inhibitory cytokines and metabolic disruptors, T_regs_ impose a terminal functional barrier within the TME to inhibit the infiltrated effector cells. However, T_regs_ also reside on the perimeter where the tumor meets the stroma (peritumor) and act as a functional and physical barrier to tumor immune infiltration.

## T_regs_ as Peritumoral Anti-Inflammatory Barriers

The non-tumor cells within the TME make up the stromal compartment and include different lineages of fibroblasts that secrete various types and amounts of extracellular matrix (ECM) proteins that influence T cells migration. Among these proteins are fibronectin (FN) and collagen (COL), with COL being more abundant in the tumor stroma and having increased stiffness which impedes T cell motility (70). T_regs_ are found in the stroma of various tumors types and correlates with poor outcome (71–74) (**Figure 1B**). Using 3D *in vitro* culture of T_regs_ in a COL gel matrices, T_reg_ markers were shown to be upregulated in high-density, compared to a low density, COL matrix, and also associated with decreased cytolytic activity (75). However, the interplay between T_regs_ and COL needs to be further defined. In a model of radiation-induced pulmonary fibrosis, T_regs_ promoted epithelium-to-mesenchymal transition (EMT) *via* β-catenin (76). In support of this, ectopic expression of Foxp3 by murine non-small cell lung cancer cells promoted EMT and tumor metastasis (77). Further studies to determine the direct role of T_regs_ in COL deposition and EMT are warranted.

IDO induces T_reg_ differentiation through the generation of tryptophan metabolites and subsequent aryl hydrocarbon receptor signaling (78, 79). IDO inhibits effector T cell activity and it has been shown in gastric cancer cell lines to be associated with ECM, COL metabolic and catabolic processes. Specifically *IDO1* and *COL12A1* synergistically promoted cell migration *in vitro* (80). In a B16 melanoma model, the IDO1 inhibitor LW106 decreased tumor-associated stromal cells and COL deposition, and increased infiltration of effector cells. Additionally, LW106 decreased T_regs_ and delayed tumor growth, suggesting a potential role for T_regs_ in LW106 efficacy, however the direct impact of LW106 on T_reg_ differentiation was undefined (81).

Fibroblasts isolated from tissue of invasive breast cancer patients had increased growth and invasion rate when treated with TGFβ, which was hypothesized to foster tumor invasion. Head and neck cancer patient-derived xenografts showed upregulation of TGFβ signaling in patients that progressed with cetuximab, an epidermal growth factor receptor inhibitor, compared to sensitive patients (82). This latter study showed elevated TGFβ1 signaling in cancer-associated fibroblasts (CAFs) in cetuximab progressors (83). In a model of pancreatic cancer, CAFs were found to express lower levels of *Col* and *Fn1* mRNA when T_regs_ were deleted, which was accompanied by an increase in effector CD4^+^ and CD8^+^ T cell infiltration, and was proposed to result from the loss of *Tgfb1* produced by T_regs_ (84). It is hypothesized that T_reg_ production of TGFβ1 promotes fibroblast differentiation into CAFs (**Figure 1B**).

Collectively, these findings suggest a role for stromal T_regs_ in the promotion of COL and CAF formation, EMT and metastasis which creates a ‘rigid’ barrier to tumor immune infiltration. Ultimately, T_regs_ support an immunosuppressive stroma, and favor metastasis and disease progression. However, the mechanisms T_regs_ utilize to execute these pro-tumor effects and the therapeutic strategies to selectively inhibit these stromal T_regs_, remain obscure.

## T_regs_ as Barriers to Tumor Infiltration by Augmenting Tumor Angiogenesis

Blood supply into the TME is critical for the survival and growth of tumors, and angiogenesis positively correlates with disease progression (85). Metabolically active tumors utilize conserved angiogenic mechanisms found in wound healing to mediate growth of new blood vessels. Hallmarks of tumor vasculature includes disorganized and immature vessels that lack vessel hierarchy and have increased permeability (86). Additionally, lymphatic vessels in the TME are dilated and leaky, which results in the accumulation of fluid and waste products. However, functional lymphatics exist at the tumor margin and are sufficient to mediate metastasis (87). The consequences of these features include metastasis and poor delivery of cancer therapies, but of interest is the inability for tumor infiltration of anti-tumor immune cells.

Tumor angiogenesis is driven by high levels of pro-angiogenic molecules, such as members of the vascular endothelial growth factor (VEGF), platelet-derived growth factor (PDGF-B) and TGFβ families, as well as hypoxia (86) (**Figure 1C**). VEGF-A is produced upon binding of the hypoxia-inducible factor 1 (HIF-1) α and β heterodimer to the *VEGF* promoter (88). VEGF-A produced by intratumoral CCR10^+^ T_regs_ in a CCL28-expressing murine ovarian tumor model, increased angiogenesis and tumor growth (30). Similarly, Helios^+^ T_regs_ in a lymphoblastic leukemia model induced angiogenesis *via* the VEGF-VEGFR2 pathway (89). VEGF-C also utilizes VEGFR2 and VEGFR3 to induce lymphangiogenesis (90). Although T_regs_ do not produce VEGF-C, the lymphatic system represents another avenue in which T_regs_ prevent proper effector T cell tumor infiltration.

Another feature of tumor-associated vessels is the ability to communicate with the immune milieu. Endothelial cells induce Fas ligand (FasL) expression upon exposure to prostaglandin E_2_ (PGE_2_), hypoxia and T_reg_-produced VEGF and IL-10 to mediate T cell apoptosis (91, 92). Endothelial FasL preferentially kills CD8^+^ T cells, while sparing T_regs_ due to T_reg_ expression of the anti-apoptotic gene, FADD-like IL-1β-converting enzyme (92) (**Figure 1C**). A feed-forward loop may exist in which VEGF-A and IL-10-producing T_regs_ in the TME promotes CD8^+^ T cell exclusion yet favors T_reg_ infiltration, which further adds to the VEGF-A and IL-10 pools.

Targeting T_regs_ through inhibition of the VEGF pathway may be advantageous as T_regs_ not only produce, but also respond to, VEGF through expression of VEGFR2 and its co-receptor Neuropilin-1 (NRP1), the latter of which is highly expressed on murine and intratumoral human T_regs_ (93–95) (**Figure 1C**). Strikingly, a NRP1 antagonist increased CD8^+^ T cell infiltration and decreased tumor growth in a murine model (96, 97). The addition of a VEGF blocking antibody to a model of adoptive cell therapy led to increased tumor infiltration of transferred cells and a reduction in tumor growth (98). Use of the immunomodulatory drug thalidomide in chronic lymphocytic leukemia decreased NRP1 expression on T_regs_, which may contribute to the reported antiangiogenic properties (99). However, efficacy of these therapies may vary depending on the organization and location of the blood vessels within and around the tumor bed. For example, location of the vasculature within the tumor, either throughout the tumor mass (tumor vessels) or within the stroma (stromal vessels), dictated the efficacy of VEGFR2-blocking antibodies, with only the former producing a significant anti-tumor response (100). In this study, stromal vessels mediated extravasation of immune cells directly to the stroma where they were trapped in the dense architecture surrounding the tumor mass, whereas tumor vessels mediated extravasation of immune cells directly to the tumor. The difference in therapeutic response may be attributed to the spatial distribution of vessels and T_regs_ and/or that this is simply reflective of a more immune-impacted tumor, which is known to be a positive prognostic indicator (101–103). Collectively, this may explain the seemingly paradoxical findings that T_regs_ may in certain circumstances appear to be a positive prognostic factor of survival

In summary, T_regs_ support pro-tumor angiogenesis in the TME *via* secretion of VEGF-A and IL-10, and expression of NRP1 (**Figure 1C**). Studies to further assess the impact of T_regs_ on the efficacy of VEGF/VEGFR inhibition/blockage and anti-NRP1, and the reorganization of the immune landscape of the TME post-therapy, will be critical to improving therapeutic response.

## T_regs_ as Barriers to Immune Cell Egress in the Stroma and Periphery

T_regs_ are also found within tumor-associated tertiary lymphoid structures (TLS), in which case the positive prognostic value of mature TLS now predicts worse outcomes and relapse in many cancer types (104–106). T_regs_ in TLS of a lung adenocarcinoma model prevented an anti-tumor response, and T_reg_ depletion resulted in increased proliferation and tumor infiltration of effector T cells (107). Similarly, CD8^+^ T cells and natural killer (NK) cells secrete IFNγ, tumor necrosis factor (TNF-α) and lymphotoxin α3, which induce neogenesis of high endothelial venules (HEV) that resemble lymph node (LN)-like vasculature and mediate T cell infiltration (108) (**Figure 1C**). A study showed that HEV formed when T_regs_ were depleted, and attributed HEV formation to increased TNF-α from T cells (109, 110) (**Figure 1C**). However, a study of colorectal cancer patients showed a positive correlation of TNF-α expression with positive LN stage and tumor recurrence (111). These studies illustrate the divergent role of lymphatics in the TME, thus more research is needed to understand the intricacies of TLS and HEV formation to therapeutically exploit their anti-tumoral role.

Tregs utilize unique mechanisms in the draining secondary lymphoid tissues to prevent recruitment to the TME. T_regs_ found in the peritumoral LN of a pancreatic ductal carcinoma model expressed CTLA-4, and CTLA-4/CD80 ligation with DCs inhibited conventional CD4^+^ T cell tumor infiltration (112). Although the mechanism is unclear, T_reg_ : DC interaction decreases CD80/CD86 expression on DCs and induces production of IDO to suppress effector function (43, 47, 68) (**Figure 1D**). Similarly, T_regs_ utilize perforin to directly kill DCs in tumor-draining LN (113) (**Figure 1D**). Altogether, T_reg_ suppression of DCs prevents effector T cell activation and lymphatic egress to the tumor site, thus promoting impaired anti-tumor immunity.

Collectively, T_regs_ in the stroma and periphery prevent tumor infiltration of immune cells by suppressing HEV formation, interfering with T cell activation by APCs and suppressing the production of proinflammatory cytokines by effector T cells. The anti-tumor effects seen with immunotherapies that block T_reg_-mediated suppression of the T cell/APC synapse and ultimately increase proinflammatory cytokines, may concurrently promote HEV formation and restructuring of the stroma, therefore the need for complimentary spatial and functional T_reg_ studies is pertinent.

## Conclusions

T_regs_ have diverse mechanisms to maintain tumor immune exclusion by affecting immune and non-immune cells, inside and outside of the tumor mass. Foundational studies interrogating intratumoral T_regs_ along with mechanisms of action for cancer immunotherapies have highlighted the impact intratumoral T_regs_ have on suppressing the anti-tumor response. However, mechanistic details of how to overcome these barriers are incomplete, leading to the following key questions:

***(1)** What is the extent of T_reg_ and stromal cell interactions, and how do these interactions impact the composition of the stroma?* Initial findings suggest that T_regs_ and stromal cells work together to prevent tumor immune infiltration via induction of CAFs by T_reg_-derived TGFβ. CAFs increase deposition of COL and FN and maintain T_reg_ suppressive functions. However, it is unclear if CAFs and T_regs_ need to directly interact for this feedback loop to occur and if other signaling events are needed to establish this suppressive peritumoral barrier. If this is a contact-dependent mechanism, it may be advantageous to develop therapeutics (i.e. blocking antibodies or inhibitors) that prevent the interaction of these two cell types within the stroma.
***(2)** What are the mechanisms that retain T_regs_ in the stoma?* CAFs support physical barriers that hinder effector T cells propagation in the stroma, where T_regs_ are abundant. Human T_H_2-like T_regs_ (GATA3^+^CCR4^+^) have the highest chemotaxis, viability and suppressive function, and are enriched in melanoma and colorectal cancer (114). GATA3 has been shown to bind to the promoter/enhancer of the IL-7 receptor and lL-7 signaling in T_regs_ is critical for development, expansion and peripheral homeostasis (115, 116). Additionally TGFβ promotes IL-7 receptor expression (117). One may then hypothesize that since CAFs produce IL-7, CAFs may support the proliferation of T_H_2-like T_regs_ in the stroma, thus maintaining an immunosuppressive stroma. Additionally, CAFs from hepatocellular carcinoma induce IDO in regulatory DCs, which promotes T_reg_ proliferation (118). Collectively, these factors may provide a stromal environment favorable to T_regs_, a notion strengthened by the observation that T_reg_-rich adenocarcinomas expressed higher TGFβ and VEGF which may reinforce T_reg_ suppressive function and stability, respectively (119). These observations support the need for further investigation into the effects of anti-TGFβ and VEGF therapies on the stromal compartment and distribution of T_regs_ throughout. However, anti-VEGF therapy in this context may be detrimental if the stroma is heavily vascularized.
***(3)** Do Tregs utilize a common pathway to promote angiogenesis and lymphangiogenesis, and can this pathway be therapeutically inhibited to normalize tumor vascularization and increase immune infiltration?* Peritumoral and intratumoral vasculature and lymphatics greatly dictates tumor infiltration of effector cells, however, specific mechanisms T_regs_ use to alter these structures is incomplete. Anti-angiogenic molecules in the clinic, such as sunitinib (receptor tyrosine kinase inhibitor) and bevacizumab (anti-VEGF), prevent the accumulation and function of T_regs_ by reducing their proliferative capacity and production of IL-10 and TGFβ, respectively (120, 121). As a VEGF co-receptor, NRP1 is a promising therapeutic as T_reg_-restricted deletion of NRP1 not only results in loss of suppressive function but also a gain of effector function via the expression of T-bet and production of IFNγ (95). The high expression of NRP1 by human tumoral T_regs_ in contrast to peripheral T_regs_ makes the VEGF/NRP1 axis a promising therapeutic target in order to normalize the vasculature and enhance effector T cell responses (122).
***(4)** Do T_regs_ utilize one suppressive mechanism preferentially to create multiple barriers to effective T cell infiltration, and if so, can this be targeted therapeutically to curtail multiple barriers of immune exclusion simultaneously?* T_regs_ suppress the anti-tumor immune response through numerous mechanisms however, there are some recurring elements that when targeted could ameliorate multiple barriers (123). Of particular interest are IDO and NRP1. IDO inhibition may recruit peripheral effector T cells and reinvigorate intratumoral effector T cells, allowing for effective immune infiltration and anti-tumor activity, respectively. IDO inhibition in the peritumoral stroma may lower COL deposition which would increase the tumor infiltration of effector T cells. NRP1 blockade may lower the suppressive function of intratumoral T_regs_ and suppress angiogenesis. Targeting IDO and/or NRP1 may promote tumor infiltration and generate a less suppressive TME.

In summary, future studies must utilize mechanistic and spatial approaches to dissect the suppressive mechanisms employed by T_regs_ at various locations in the TME. These spatially-mapped functional studies will aid in the development of novel immunotherapies that aim to dismantle the T_reg_-induced physical, metabolic and trafficking barriers within the TME.

## Author Contributions

ES, AG, CW, and DV wrote the article. All authors contributed to the article and approved the submitted version.

## Funding

This work was supported by the National Institutes of Health [F32 CA247004 to AG, T32 CA082084 to AG and ES; P01 AI108545, R01 CA203689 and P30 CA047904 to DV].

## Conflict of Interest

DV: cofounder and stockholder – Novasenta, Tizona and Trishula; stockholder – Oncorus, Werewolf and Apeximmune; patents licensed and royalties - Astellas, BMS; scientific advisory board member - Tizona, Werewolf, F-Star, Bicara, Apeximmune; consultant - Astellas, BMS, Incyte, Almirall, G1 Therapeutics; research funding – Novasenta, BMS and Astellas.

The remaining authors declare that the research was conducted in the absence of any commercial or financial relationships that could be construed as a potential conflict of interest.
